# Population dynamics, structure and behavior of *Anopheles darlingi *in a rural settlement in the Amazon rainforest of Acre, Brazil

**DOI:** 10.1186/1475-2875-10-174

**Published:** 2011-06-24

**Authors:** Paulo Rufalco Moutinho, Luis Herman Soares Gil, Rafael Bastos Cruz, Paulo Eduardo Martins Ribolla

**Affiliations:** 1 Departamento de Parasitologia, Instituto de Biociências de Botucatu, Universidade Estadual Paulista, Botucatu, São Paulo, Brasil; 2 Instituto de Pesquisa em Patologias Tropicais, Porto Velho, RO, Brasil

## Abstract

**Background:**

*Anopheles darlingi *is the major vector of malaria in South America, and its behavior and distribution has epidemiological importance to biomedical research. In Brazil, *An*. *darlingi *is found in the northern area of the Amazon basin, where 99.5% of the disease is reported.

**Methods:**

The study area, known as Ramal do Granada, is a rural settlement inside the Amazon basin in the state of Acre. Population variations and density have been analysed by species behaviour, and molecular analysis has been measured by ND4 mitochondrial gene sequencing.

**Results:**

The results show higher density in collections near a recent settlement, suggesting that a high level of colonization decreases the vector presence. The biting activity showed higher activity at twilight and major numbers of mosquitos in the remaining hours of the night in months of high density. From a sample of 110 individual mosquitoes, 18 different haplotypes were presented with a diversity index of 0.895, which is higher than that found in other *Anopheles *studies.

**Conclusions:**

*An. darlingi *depends on forested regions for their larval and adult survival. In months with higher population density, the presence of mosquitoes persisted in the second part of the night, increasing the vector capacity of the species. Despite the intra-population variation in the transition to rainy season, the seasonal distribution of haplotypes shows no change in the structure population of *An. darlingi*.

## Background

Malaria is one of the most important tropical diseases in the world. WHO data report a total of 106 malaria-endemic countries and 151 million estimated cases in 2009 [1]. In South America, there are a high number of disease notifications in Brazil, Colombia, Peru, Venezuela, Suriname and Bolivia. These countries have large tracts of Amazon rainforest, South American biome and habitats for many *Anopheles *species that have high potential to be malaria vectors [2,3].

Brazil has the largest number of malaria cases and malaria-related deaths in the Americas, and 15% of its population lives in at-risk areas, which are concentrated in the states of the Amazon Basin, with an average of 500 thousand notifications per year [4]. The strategies and targets for malaria control include diagnosis, disease treatment and prevention by mosquito control. Therefore, understanding the biology and behaviour of the vector is extreme importance for efficient control of the disease.

The main vectors of malaria in South America are: *Anopheles albimanus*, *Anopheles darlingi *and *Anopheles nuneztovari *in Colombia [5,6]; *An. darlingi *and *Anopheles benarrochi *in Peru [7,8]; *An. darlingi *in Bolivia [9]; *An. darlingi, Anopheles marajoara *and *Anopheles aquasalis *in Venezuela [10-12] and *An. darling *and *An. aquasalis *in Brasil [13,14]. The presence of *An. darlingi *in the countries cited explains why it is the major target of most studies of malaria vector dynamics in the continent.

*Anopheles darlingi *is the major vector of malaria in Brazil. There are two main factors that may have contributed to this ability: the species is highly susceptible to the *Plasmodium *sp. that infect humans [15] and demonstrates anthropophilic behaviour [2,16]. With respect to its biology and development, the larvae utilizes commonly water reservoirs close to houses as breeding grounds: lakes, margin rivers, streams and flooding areas, which are shaded or partly shaded, and mats of floating debris and vegetation [17,18]. Human presence in the Peruvian Amazon influences the creation of new breeding sites via impoundment and creation of large lakes for fish farming [19].

Concerning seasonality, models differ according to the area and their breeding pattern: riverines areas (dwelling beside a river) with low anthropogenic action show low *An. darlingi *densities during the dry season, which increase a few months after the beginning of the rainy season, reaching their highest levels at the peak of this season. For inland areas with high anthropogenic action, where there is a greater presence of artificial breeding sites, the water reserve tends to retain its capacity during the dry seasons and the mosquito densities rise to high values by the end of the rainy season, persisting at a high level in the dry season [14,20-22]. Tadei [23] reported that low-lying and flooded areas have numerous breeding sites and high mosquito densities, and the dry and rainy seasons do not have much effect on anopheline populations. Finally, some studies still show that there are no correlations between *An. darlingi *populations and rainfall, so this result might be a consequence of the known variability in mosquito abundance among localities [11,12].

The periods of biting activity are crepuscular and overnight, with peaks in the early hours of the evening; there may be an extension of this activity during the night, according to the season and to the vector's population density [3,24,25]. As for endophilic/exophilic behavior, it is hypothesized that this feature changed because of the introduction of control methods, which consist of the use of indoor insecticides [26]. Early analysis of *An. darlingi *showed the species to be endophilic, as after feeding on blood, the females rested in the internal structure of the house [27,28]. Recent studies show a behavior change, where there was an increased presence of mosquitoes in the peri-domicile, which is located outside the house but close to it [3,14,25]. New prophylactic measures are being studied, as is the use of repellents and insecticide impregnated bed nets [29,30].

Various factors that change the habitat and performance of *An. darlingi*, mainly due to human interventions, have led us to research an intraspecies difference between the populations. Forest degradation, increased housing in the local forest and climate changes are strong influences on *Anopheles *populations. Therefore, it is necessary to obtain tools to identify and characterize the intraspecies variability in *An. darlingi*.

For studies with *An. darlingi*, which has an extensive geographic distribution, it is necessary that the marker chosen provides a degree of polymorphism detectable among individuals. The molecular marker contributes to the understanding of the heterogeneity of the species, clarifying the important information about the vector and its population structure [31,32]. Wide use of mitochondrial DNA (mtDNA) sequencing determines population structure in medical entomology [33-35]. The mitochondrial genome consists of a small, circular molecule with conserved genetic content (only 37 genes) and a simple genomic structure (maternal inheritance, absence of recombination, small or absent intergenic areas, absence of introns, repetitive DNA, pseudogenes and transposable elements) [36]. The molecule evolves about 5-10 times faster than the nuclear genes do [36-38]. Constantly used in studies with *Aedes aegypti *[39-41], this marker showed satisfactory results with *An. darlingi *[42], which were high polymorphism and similar indexes of nucleotide diversity from other mitochondrial genes for neotropical anophelines [43,44].

The state of Acre is situated in the Amazon basin. In 2008, 9,410 malaria notifications were reported, with 8,595 occurring in rural settlements [4]. The study site of this paper, the municipality of Acrelândia, represents a risk area [45] and is the subject of several publications on the *Anopheles *sp. [46-48]. The aim of this study was to verify the behavior, distribution and population structure of the major vector of the region, *An. darlingi*. The area is subject to continually gradual changes of the geographic space, and this fact highly influences *An. darlingi *dynamics.

## Methods

### Study Area

**Figure 1:**
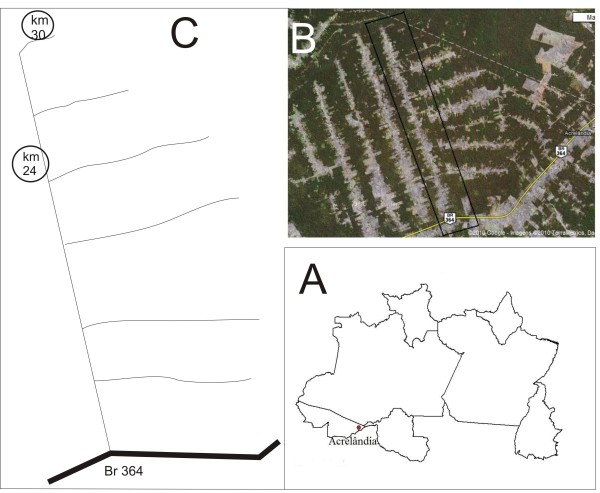
**Study Area**. A: Acrelândia, in northern Brazil. B: satellite image from "Ramal do Granada" (^©^2010 Google-Images ^©^2010 TerraMetrics, Nasa, Dados cartográficos ^©^2010 Maplink). C: "Ramal do Granada" with the sampling sites km 24, km 30 and BR 364 (Point 0).

Acrelândia, 122 km from Rio Branco, is a small town with approximately 12,000 inhabitants located in the western state of Acre (Figure 1). The area has a history of urbanization like other regions of the Amazon biome, starting with the Rubber Boom in the early twentieth century, followed by other extractive activities such as the mining and lumber industries. Currently, agriculture is the main factor responsible for the continued deforestation.

The *Ramal do Granada *(Brazilian Federal Highway 364) is located in rural area of Acrelândia, and belongs to the Pedro Peixoto Agricultural Settlement Project, a Settlement Program Directed (*Programa de Assentamento Dirigido *- PAD) responsible for subdivision and land distribution in various uninhabited regions of the Brazil. The *Ramal do Granada *(Figure 1) has a linear extension of 30 km from point 0 (BR 364-highway) and includes households along an unpaved road, with the economy based on agriculture, mainly with livestock. The number of homes and the degree of deforestation vary according to the position along the road: the proportion of degradation that correlates with human actions can be assessed via the linear distance from point 0. The tendency is this; the farther from point 0, the lower the number of residents and the level of deforestation.

**Figure 2:**
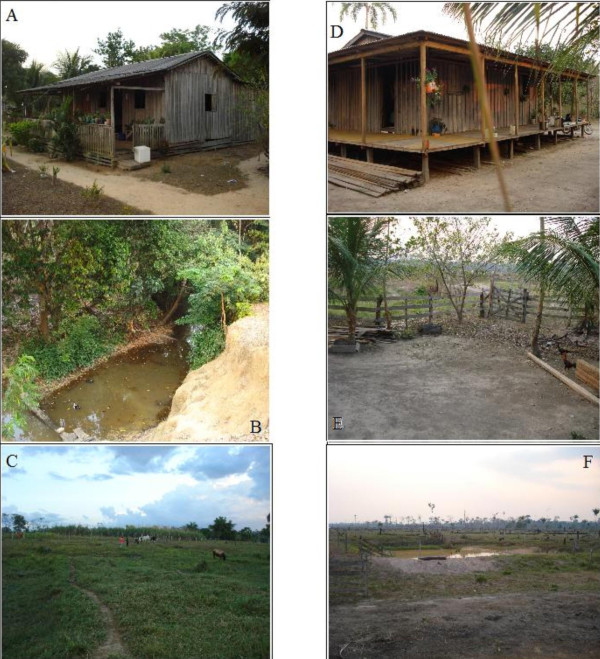
**Collection points**. Km 24 (A: indoor capture, B; outdoor capture, C: pasture behind the house) and Km 30 (D: indoor capture, E; outdoor capture, F: pasture behind the house).

Considering this information, two sampling sites for *Anopheles *adult were selected from *Ramal do Granada *(Figure 1): km 24 (455ft; S09°44.542'; WO67°07.212') and km 30 (421 ft; S 09°41,247'; WO67°07,692). The km 24 represents the area with more degradation and higher numbers of houses, and km 30 represents the area with more recent occupation. Both sites have the following: streams and water reserves; flooded areas; similar structure of the houses; and the presence of riparian forests and pasture near the residences (Figure 2).

### Mosquito collection

Each mosquito collection consisted of indoor and outdoor capture, composed of three consecutive days; 3-hour captures at first and second day (km 24 and km 30 respectively) and 12-hour capture at the third day (only km 30). The 3-hours capture occurred between 6:00 pm and 9:00 pm and the 12-hours capture between 6:00 pm and 6:00 am. The interval between 6:00 pm and 9:00 pm for the 3-hour capture was decided upon by consulting the literature, which reported the twilight behaviour of *An. darlingi *[3,24,25,49].

The weather conditions specific to each collection day are listed in Table 1. Temperature and RAH are the average values from two digital thermo-hydrometers ITHT-2000 (Instrutemp), located indoor and outdoor capture. The rainfall values were obtained with the State Civil Defense of Acre. Moon phases were collected in http://www.moon-phases.net and the climatic condition were noted during capture day. There was a concern with the choices of the capture day, avoid events that change the biting activity (rain, fogs, full moon), establishing a pattern of weather conditions for the collections.

**Table 1:** Weather Conditions at the collection points (km 24 and km 30) during the work period.

Month	Capture day	Temperature	(C°)	RAH (%)	Rainfall (mm)	Moon Phases	Climatic Condition
May/08	3h-km24	24.4		85	0	New Moon	Clean
	3h-km30	27.3		79	0	Waxing Crescent	Clean
	12h-km30	23.1		90	0	Waxing Crescent	Clean

July/08	3h-km24	25.3		80	0	Waxing Crescent	Clean
	3h-km30	24.8		68	4.9	Waxing Crescent	Partly cloudy
	12h-km30	22.7		80	0	First Quarter	Partly cloudy

Sept/08	3h-km24	27.2		70	0	Waxing Crescent	Clean
	3h-km30	28.8		57	0	Waxing Crescent	Clean
	12h-km30	25.6		81	0	Waxing Crescent	Clean

Nov/08	3h-km24	23.2		97	7.2	Waning Crescent	Partly cloudy
	3h-km30	28.1		79	0	Waning Crescent	Partly cloudy
	12h-km30	24.5		95	8.6	New Moon	Cloudy

Feb/09	3h-km24	28.9		84	0	Waxing Crescent	Partly cloudy
	3h-km30	28.7		74	25.3	Waxing Crescent	Partly cloudy
	12h-km30	26.4		88	83.5	First Quarter	Cloudy

Temperature and RAH; average values from two thermo-hydrometers, located indoor and outdoor capture; Rainfall: State Civil Defense of Acre; Moon Phases: http://www.moon-phases.net; Climatic Condition: noted during capture day.

The mosquitoes was captured using human bait, performed by the own authors. Following the seasonal variation of rainfall, five collections were conducted over ten months: May, July, September and November 2008 and February 2009. Mosquitoes were identified using a key species of the subgenus of *Nyssorhynchus *[50] and preserved in 70% isopropyl alcohol.

### Extraction, Amplification and Purification of mtDNA

For the extraction of mtDNA from *An. darlingi*, Chelex^® ^Molecular Biology Grade Resin (Bio-Rad Laboratories) was used. DNA was extracted from mosquitoes individually, following the manufacturer's recommendations. The extracted DNA was quantified using an ND-1000 (Nanodrop) spectrophotometer and its purity was also checked.

Amplification reactions were conducted according to the protocol of Gorrochotegui-Escalante with modifications [51]. Amplification was done in a final volume of 40 ul, using 2 ul of the DNA sample, 20 uL Gotaq Colorless Master Mix (Promega), 12 uL ultrapure water (Aster) and 6 uL of oligonucleotides specific for the ND4 gene: 3uL of ND4-F (5'-TGATTGCCTAAGGCTCATGT-3') and 3uL of ND4-R (5'-TTCGGCTTCCTAGTCGTTCAT-3') (Invitrogen). Amplification reactions were performed in a Whatman Biometra (T Gradient) thermocycler with temperature cycles of: three cycles of 94°C/2 min, 37°C/2 min, and 72°C/1 min, followed by 35 cycles of 94°C/30 s, 50°C/30 s, and 72°C/1 min, with a final extension cycle of 72°C/5 min. Polymerase chain reaction (PCR) products were subjected to electrophoresis through an agarose [52]. The specific region of the ND4 gene that was amplified corresponds to nucleotides 8.519-8.880 in *Aedes albopctus *[GenBank:AY072044], and has been used in other publications by our research group [40,42].

PCR products were purified using Montage PCR Centrifugal Filter Devices (Millipore), following the manufacturer's recommendations.

### Sequencing mtDNA and Data Analysis

The purified PCR products and an aliquot of the oligonucleotides specific for the ND4 gene (10 pmol/L) were sent to the bio-molecular company Macrogen (Seoul, South Korea). Sequencing was conducted under BigDye Terminator Cycling Conditions. The reaction products were purified using ethanol precipitation and run using the Automatic Sequencer 3730XL. ND4 gene sequences were analysed, as was the reaction efficiency of sequencing. A fragment of 286 base pairs was used to compare the sequences and for further analysis. These sequences were aligned using Clustal W [53], and misaligned nucleotides were manually adjusted. The program MEGA [54] was used to perform phylogenetic and molecular analysis. Distance (Neighbor Joining, NJ) and parsimony methods were used to construct phylogenetic trees [55] using the Kimura 2 parameters distance [56]. Nucleotide sequences and haplotype frequencies were calculated using DnaSP version 3.5 [57]. Genetic analysis of population differentiation, haplotype diversity index, and nucleotide diversity were calculated with Arlequin 3.0 software [58] and a haplotype map was constructed by TCS, version 1.12.

## Results

### Mosquito Collections

A total of 3,486 mosquitoes were collected, with 2,982 (85.5%) being *An. darlingi*. Others *Anopheles *species were also collected: *Anopheles deaneorum*, *Anopheles rangeli*, *Anopheles albitarsis, Anopheles braziliensis, Anopheles triannulatus *and *Anopheles argyritarsis*. Table 2 depicts the distribution of *An. darlingi*, only for the 3-hour capture. The collections show significant quantitative differences between the sites: km 24 showed a lower density of *An*. *darlingi *compared to 30 km, and the outdoor capture had the major number of mosquitoes collected. The low value of the sample at km 24, compared to 30 km, was observed for all of the months.

**Table 2:** *An. darlingi *densities during the 3-hour captures.

	Km 24		Km 30	
Month	Indoor	Outdoor	Indoor	Outdoor
May/2008	4 (1.3)	1 (0.3)	66 (22)	102 (34)
July/2008	0	5 (1.7)	2 (0.7)	145 (48.3)
September/2008	0	5 (1.7)	0	7 (2.3)
November/2008	0	1 (0.3)	8 (2.7)	37 (12.3)
February/2009	2 (0.7)	3 (1)	95 (31.7)	399 (133)

Total	6	15	171	690

Total number collected per location (number of mosquitoes/person/hour).

**Figure 3:**
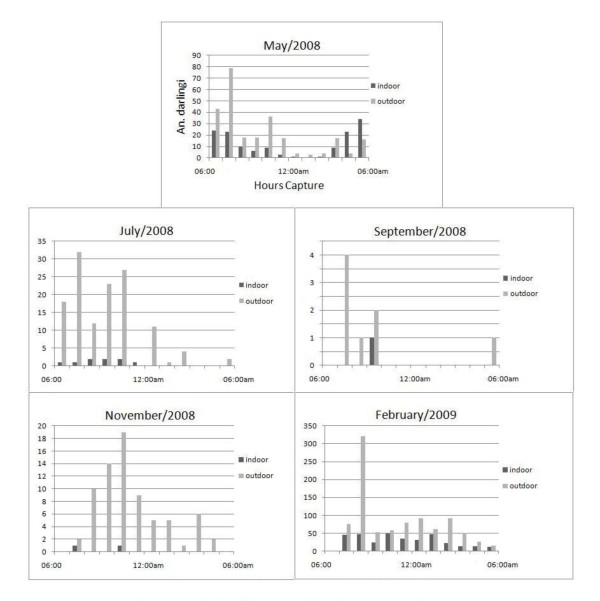
***An. darlingi *12-hour capture**. Biting activity of *An. darling *in 12-hour capture at km 30.

The 12-hour captures shows a higher concentration of *An. darlingi *at the first part of the night, with biting activity starting at sunset. Figure 3 shows a pattern of biting activity that alternates according the population density. May 2008 indoor capture displayed a classic bimodal cycle. For the May, July, September and November outdoor captures, the occurrence is predominantly in the first part of the night. February 2009, month with the highest population density, registered biting activity until the early morning hours in both captures. 1,899 *An*. *darlingi *were collected in the twelve-hour capture, with 73.6% being outdoor captures.

### Seasonal Variation

**Figure 4:**
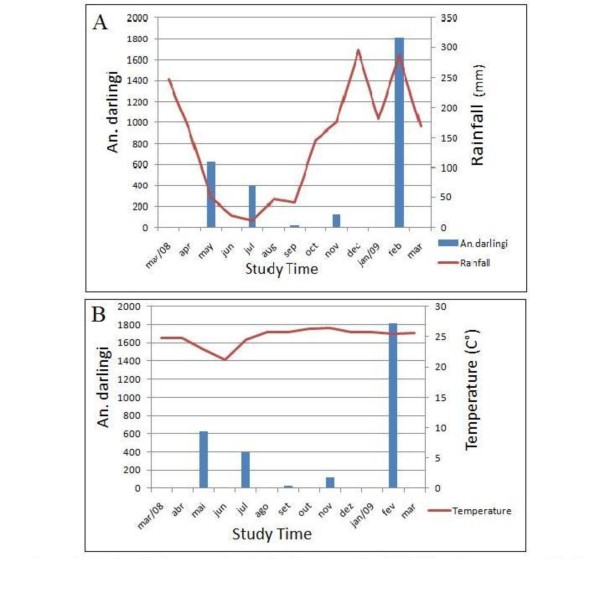
***An. darlingi *seasonal density; rainfall (A) and temperature (B)**. The values correspond the monthly averages.

Figure 4 represents the distribution of the anopheline mosquitoes relative to precipitation and temperature monthly average. Each month's collection represents the total number of *An. darlingi *captured from both sites in three days (3-hour capture at km 24, 3-hour capture at km 30 and 12-hour capture) and shows the variation of mosquitoes according to the study period. It is important to note this change; May to July 2008 there was an observed decrease of precipitation, which followed the decrease of *An. darlingi*; opening of the dry season and diminution of temperature. September 2008 is the month that best illustrates how the mosquito density decreases during the dry season.

With the increase in precipitation and the rise in temperature marking the beginning of the rainy season. November 2008 reflects this transition with a significant increase of *An*. *darlingi *in relation to September. Lastly, February 2009, two months after the high record for rain precipitation, showed the largest number of mosquitoes, representing approximately 61% of the entire collection.

### Sequencing analysis of mtDNA

One hundred ten mosquitoes were sequenced: 30 from July, 12 from September, 33 from November and 35 from February. Only *An. darlingi *indoor and outdoor from km 30, the collection site that shows higher density, were chosen, thereby obtaining significant values for the seasonal variation of species. The alignment of these sequences showed the presence of 20 polymorphic sites representing 18 different haplotypes. The populations showed an index of nucleotide diversity of π = 0.0127, and a haplotype diversity Hd = 0.895. A total of 20 mutations were identified, with 16 synonymous mutations.

**Figure 5:**
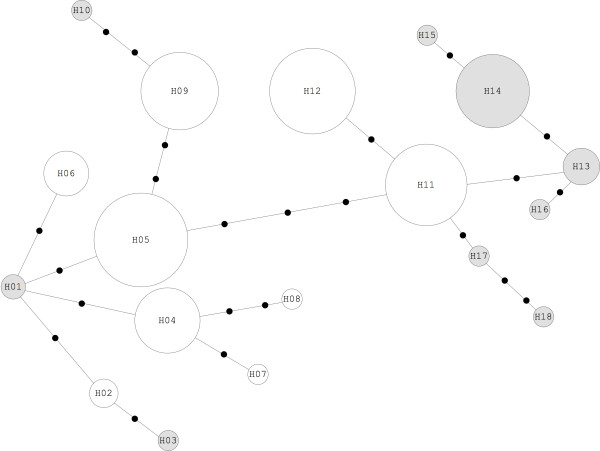
**Statistical parsimony network**. 18 haplotypes of *An. darlingi *populations for ND4 mitochondrial fragments. Black dots along the lines indicate mutational steps between haplotypes (unrepresented in the sample). The size of the sphere is proportional to the number of individuals carrying the haplotype.

Analysis of sequences and the map of haplotypes by TCS (Figure 5), indicate the occurrence of two major groups, separated by two mutational steps. Table 3 aggregates the sazonal variation of haplotypes with samples numbers, between July, 2008 and February, 2009. There is a difference between the haplotype frequencies in each month: five exclusive haplotypes in November 2008 (H3, H8, H16, H17 and H18) are not found in the collection for February, 2009, the month that had the highest density of *An. darling*. The haplotypes with the highest numbers of mosquitoes (H4, H5, H9, H11 and H12) are present in all months of collection, except H6, with only six samples. September, 2008, the month with the lowest density and all *An. darlingi *collected were analyzed, presented seven haplotypes. Six of these are found in the previous month and remained in the population until February. Pair-a-pair Fst comparisons between populations (Table 4) shows that November and February (Fst = 0,05, P < 0,01) is the only significant result.

**Table 3:** Temporal variation of haplotypes.

Month/Haplotype	Jul/08	Sep/08	Nov/08	Feb/09	number of individuals
H1	XXXXX			XXXXX	2

H2				XXXXX	3

H3			XXXXX		1

H4	XXXXX	XXXXX	XXXXX		11

H5	XXXXX	XXXXX	XXXXX	XXXXX	18

H6	XXXXX	XXXXX	XXXXX	XXXXX	6

H7				XXXXX	1

H8			XXXXX		1

H9	XXXXX	XXXXX	XXXXX	XXXXX	14

H10	XXXXX				1

H11	XXXXX	XXXXX	XXXXX	XXXXX	15

H12	XXXXX	XXXXX	XXXXX	XXXXX	16

H13		XXXXX			4

H14	XXXXX		XXXXX	XXXXX	13

H15	XXXXX				1

H16			XXXXX		1

H17			XXXXX		1

H18			XXXXX		1

Exclusive haplotipe	1	1	5	2	

Cells with "XXXXX" represent the presence of the specific haplotype at that period.

**Table 4:** *F*-Statistics based on pairwise estimates of ND4 haplotype frequencies.

Month	July/2008	September/2008	November/2008	February/2009
July/2008	0.00000	-	-	-
September/2008	-0.01554	0.00000	-	-
November/2008	-0.00756	0.00131	0.00000	-
February/2009	0.00921	-0.02740	0.04663*	0.00000

*(P = 0.008)

## Discussion

The results presented here show variations in the density of mosquitoes between both sampling sites, showing a relationship between local anophelines and degraded natural areas. These changes result in several consequences for the mosquitoes fauna, establishing different models of population dynamics of malaria vectors. Some studies shows that modifications in natural environments promote succession of faunas and changes the species adaptability in urban landscapes [59-61] and these are important factors in the epidemiology of tropical diseases, being that settlement areas have growth in the Amazon basin [62].

The species diversity and the total amount of anophelines show km 30 to have major abundance and population density, while km 24 reports low density. The choice of these two sampling sites in *Ramal do Granada *was justified by similar forms of human occupation, but with different degrees of deforestation and human actions. The landscape features of km 30 are newly deforested areas with recent occupation, with a great presence of burn forest residues in pastures, and significant coverage of the Amazon rainforest. The distributions of houses in this site are similar: all are close to the road, however distant from each other, as a consequence of a lower number of residences. Unlike at km 30, the km 24 location has residences that are more aggregated and that have been occupied for longer. Collections and reserves of water are similar in both sites, with streams and artificial ponds used for raising cattle. Thus, in comparing the five 3-hour captures, it appears that differences between both sampling sites influence the development of the respective *Anopheles *population. The forest is directly related to the biology of *An. darlingi*, and this difference is linked with the ecology of the species in the environment of each site [21,63]. Studies in rural settlements in Rondônia reported, using the degree of urbanization, environment and economic activity, highest malaria risk in areas are around and next to forests [64].

The influence of human actions on populations of *An. darlingi *in *Ramal do Granada *shows that a significant level of degradation decreases the presence of this vector. The area of the most recent occupation and closer to undisturbed forest, the km 30, shows an increase in the abundance of anopheline mosquitoes compared to km 24. Epidemiological data of malaria in *Ramal do Granada *indicate that km 30 is the locality with the most susceptible hosts and the highest incidence rate of disease, associating the observations that newcomers tend to settle in forest fringes and tend to have no malaria immunity [65,66]. Malaria prevalence in Granada is similar to that described in peri-urban and rural populations in the State of Rondônia; this area consists predominantly of migrants who settled there at least a decade previously [67].

*An. darlingi *depends on the regions with forests for their larval and adult survivorship, and profound changes in their habitat may restrict their presence [68,69]. Environmental analysis and characterization of the breeding sites of both sampling sites has produced more data for this argument [19]. However, differences in the vectors' densities along the same line of a rural settlement can be affirmed analyzing these results; this is an effective tool to indicate where case monitoring and vector control should be directed [70].

The night biting activity seen in the 12-hour capture reports the trend observed in other *An. darlingi *studies in Amazon Basin; beginning at twilight, with peaks concentrated in the first part of the night [3,8]. May and July, the biting activity starts at 6:00 pm, while the others months starts after one hour (7:00 pm). The main variations verified during each sampling month were related to anopheline density; in the months with higher densities, the presence of *An*. *darlingi *persisted through the second part of the night until dawn, as shown in February. The outdoor capture in May show two peaks in the first part of the night, and indoor capture displays a bimodal pattern. The presence of more than one peak during the twilight hours is reported in outdoor capture in July, transition between the rainy and dry season, and outdoor capture in November, transition between the dry to rainy season. July density concentrated in the first part of the night, and November peaked later (11:00 pm), with activity until 5:00 pm. This variations showed that though *An. darlingi *is most active at twilight, a prolongation of biting activity is common outside the standard time range, which increases the vector capacity of the species. The 12-hour capture also showed a high difference between indoor and outdoor mosquitoes.

The seasonality data is linked with the presence and types of breeding sites in the region, and is consequence of the extent of landscape changes. Analyzing Figure 4, indicates that the density of *An. darlingi *from May, 2008 to July, 2008 is in decline, showing a probable relationship with the decrease of rainfall and the beginning of dry season [71]. One can speculate that the decrease in precipitation left the breeding sites still able to support breeding, but not enough to maintain high densities of the vector. This prominent decrease in rain level was reflected in minimal rates for mosquito populations in September, 2008. With the transition to the rainy season, there was a considerable density increase in November, 2008, followed by a large growth in February 2009, 61% of the total *An. darlingi *collected during the all study. Similar data in two villages located in Maroni River (Suriname and French Guiana frontier) shows after heavy rainfall providing temporary breeding places for the *A. darlingi*, which explains the rapid increase in population densities after the rain [30]. It is also important to mention that the numbers of *An. darlingi *listed in Figure 4 are the three captures aggregate (3-hour capture km 24, 3-hour capture km 30 and 12-hour capture), so that a greater proportion of these mosquitoes belong km 30. Therefore, we can assume that the natural breeding site's capacity is maximized during the rainy season [14]. However, to support this hypothesis, there is the need to perform a larval study on the breeding sites in the area and to correlate this with abundance of *An. darlingi *and river levels [12].

The choice of samples for the molecular analysis was based on two criteria: comparison between indoor and outdoor mosquitoes, and variability and seasonal distribution of haplotypes. For this, a sample selection was made, covering a significant portion of *An. darlingi *of all times in 12-hours captures. In the final methodology, were not processed *A. darlingi *from km 24 sampling site and from May, 2008. No significant variability was observed between the indoor and outdoor populations (P = 0.265), despite the fact that Forattini [72] postulated that exophilic and endophilic behavior could be a possible characteristic of distinct populations. Both collections, 3-hour and 12-hour capture, showed a prevalence of outdoor mosquitoes. The *Ramal do Granada*, as in other regions of the Amazon rainforest, has utilized control measures for malaria vectors, consisting of the use of indoor insecticides [26]. House-spraying by SESACRE (Acre State Secretariat of Health and Sanitation) is common; periodically, at three month intervals and immediately after a positive case is notified (called *Bloqueio*). Spraying occurs in the homes of patients, ridding the inside of their residence of the vectors. Quantitative data show that there is a difference between the populations of *An. darlingi *that feed in the home and those outside, as a consequence of the constant use of house spraying. However, we can observe a significant increase in biting activity in the intra-domicile region during the rainy season.

The variability in seasonal distribution of haplotypes shows monthly changes in population structure of *An. darlingi*. Were defined as exclusive haplotypes the strains found only one month of the study, and common haplotype found in two months or more. The haplotypes reported are representative samples from 12-hour capture; July, September, November 2008 and February 2009. Analyzing Figure 5 and Table 3, the two major groups, separated by two mutational steps, have the same proportion of haplotypes exclusive and common. The first group, H1 to H10 (58 samples) have four common haplotypes (51 samples) and six exclusive haplotypes (7 samples). The second group, H11 to H18 (52 samples), have three common haplotypes (44 samples) and five exclusive haplotypes (8 samples). November, transition to the rainy season and growing of *An. darlingi *population, have the greatest haplotype variability compared to other months of study.

ND4 polymorphism shows a intra-population variation regulated by environmental (rainfall, temperature, weather conditions) and anthropic factors (fogs, insecticides, forrest degradation). The Fst is significant only between November and February and reveals two distinct moments of the *An. darlingi*; the transition to the rains, having a population growth and filling of vacant niches in November and the population reaching the high density in February. This shows variation during population dynamics in transition to the rains, however suggest no shifting population structural; 84 samples from exclusive haplotypes found in July are also reported in February, which represents 76.36% of the total samples. The seasonal distribution of haplotypes shows no change in the population of *An. darlingi *in *Ramal do Granada*.

This feature of the local anopheline population is relevant because frequent changes of the landscape can affect population dynamics, allowing for the spread of groups of genes related to malaria transmission, which enhance the mosquitoes' status as vectors [31]. It is also important to mention that the *Plasmodium vivax *populations circulating in the *Ramal do Granada *are extraordinarily diverse, factor that contributes to malaria transmission in endemic regions [73].

## Conclusions

At the outset, *An. darlingi *is the major vector found in this study area. Their distribution is related to forests areas, and their abundance occurs in areas of recent colonization. *An. darlingi *behavior was similar to that described in the scientific literature: activity at twilight that persisted until halfway through the night and was exophilic. Prolongation of the biting activity was reported in months with higher densities. Variation of the population size is related to rainfall, and is a consequence of the anthropogenic factors in the region. A high density of species was reported during the rainy season. Sequencing analysis of the mitochondrial gene ND4 shows that the seasonal variability haplotype indicates a stable population, without the insertion of new strains in transition between the dry and rainy seasons.

## Authors' contributions

PRM: prepared the initial project, participated in the collections and travel to study area, development and molecular analysis of samples and drafted the manuscript. LHSG: participated in the collections and travel to study area, identification and preservation of material. RBC: participated in the collections and travel to study area, identification and preservation of material. PEMR: prepared the initial project, participated in the collections and travel to study area and molecular data analysis, as well as helped to draft the manuscript. All authors read and approved the final manuscript.
